# Economic burden of work injuries and diseases: a framework and application in five European Union countries

**DOI:** 10.1186/s12889-020-10050-7

**Published:** 2021-01-06

**Authors:** Emile Tompa, Amirabbas Mofidi, Swenneke van den Heuvel, Thijmen van Bree, Frithjof Michaelsen, Young Jung, Lukas Porsch, Martijn van Emmerik

**Affiliations:** 1 IWH (Institute for Work & Health), 400 University Avenue, Suite 1800, Toronto, Ontario M5G 1S5 Canada; 2 TNO (The Netherlands Organisation for Applied Scientific Research), Hague, Netherlands; 3 VVA (Valdani Vicari & Associati), Milan, Italy

**Keywords:** National-level, Underestimation, Incidence, Bottom-up, Stakeholders, Attributable fractions, Eurostat

## Abstract

**Background:**

Estimates of the economic burden of work injuries and diseases can help policymakers prioritize occupational health and safety policies and interventions in order to best allocate scarce resources. Several attempts have been made to estimate these economic burdens at the national level, but most have not included a comprehensive list of cost components, and none have attempted to implement a standard approach across several countries. The aim of our study is to develop a framework for estimating the economic burden of work injuries and diseases and implement it for selected European Union countries.

**Methods:**

We develop an incidence cost framework using a bottom-up approach to estimate the societal burden of work injuries and diseases and implement it for five European Union countries. Three broad categories of costs are considered—direct healthcare, indirect productivity and intangible health-related quality of life costs. We begin with data on newly diagnosed work injuries and diseases from calendar year 2015. We consider lifetime costs for cases across all categories and incurred by all stakeholders. Sensitivity analysis is undertaken for key parameters.

**Results:**

Indirect costs are the largest part of the economic burden, then direct costs and intangible costs. As a percentage of GDP, the highest overall costs are for Poland (10.4%), then Italy (6.7%), The Netherlands (3.6%), Germany (3.3%) and Finland (2.7%). The Netherlands has the highest per case costs (€75,342), then Italy (€58,411), Germany (€44,919), Finland (€43,069) and Poland (€38,918). Costs per working-age population are highest for Italy (€4956), then The Netherlands (€2930), Poland (€2793), Germany (€2527) and Finland (€2331).

**Conclusions:**

Our framework serves as a template for estimating the economic burden of work injuries and diseases across countries in the European Union and elsewhere. Results can assist policymakers with identifying health and safety priority areas based on the magnitude of components, particularly when stratified by key characteristics such as industry, injury/disease, age and sex. Case costing can serve as an input into the economic evaluation of prevention initiatives. Comparisons across countries provide insights into the relevant performance of health and safety systems.

**Supplementary Information:**

The online version contains supplementary material available at 10.1186/s12889-020-10050-7.

## Background

Information on the economic burden of work injuries and diseases is vital for policymakers attempting to allocate scarce resources to priority areas in the occupational health and safety policy arena. Over the last few decades, several researchers have estimated the economic burden of work injuries and diseases around the world [1–7]. Leigh estimated that the cost of work injuries and diseases in the United States is about US $250 billion or 1.8% of Gross Domestic Product (GDP) [2]. The Health and Safety Executive (HSE) estimated the cost of work injuries and diseases in the United Kingdom at £14 billion, about 1% of the GDP [3]. Safe Work estimated the costs of work injuries and diseases in Australia at AUS $61 billion, or 4.8% of GDP [4]. In a study of Singapore, the total cost was estimated at SG $10.45 billion, or 3.2% of GDP [5]. The International Labour Organization estimated that 4% of global GDP is lost due to work accidents and diseases [6]. A recent project by the European Agency for Safety and Health at Work (EU OSHA) found that the burden of work injuries and diseases is 3.9% of global GDP and 3.3% of European GDP [7].

Despite multiple published studies on the economic burden of work injuries and diseases, identifying robust and comparable estimates of the total burden at the country level still remains a complex undertaking, largely due to the lack of standardized methodology and uniformity in national-level data. This makes comparability across countries less reliable, and repeated measures within a country over time a challenge. Furthermore, most of the previous studies have focused on a limited number of cost subcategories, primarily labour productivity and output losses. Consequently, the true societal and economic burden of work injuries and diseases, including intangible costs, remain unknown.

Estimation of the economic burden of work injuries can be traced back to the pioneering work of Herbert Heinrich from the early twentieth Century [8], when he estimated a fraction of the economic burden of work injuries using insurance-based models (which relied on the wage cost of absenteeism). He described the non-wage cost of the economic burden of absenteeism as the “hidden part of an iceberg.” This early work gave rise to the notion of direct and indirect costs incurred by employers and the idea that a multiplier could be used as a short cut to approximate the indirect portion [9].

Over time, attempts to itemize, value, and sum the hidden part of the economic burden of work injuries and diseases turned to accounting ledgers that identified financial outlays associated with work injury and disease absences such as healthcare expenses, worker replacement costs, etc. [10] The methodology of such studies has gradually shifted from accounting for hard costs towards a broader economic impact approach that includes less tangible costs, e.g., implicit or opportunity costs in equivalent monetary terms, such as presenteeism (working with less effectiveness) [11], home production losses [2], productivity losses associated with permanent impairment [12], and staff-turnover costs (i.e., loss of skilled staff) [4].

In summary, the main differences in the methodology of economic burden of work injury and disease studies can be attributed to differences in,

- the cost estimation model used, i.e., bottom-up [2–5] versus top-down [7]
- case estimation approach, i.e., prevalence approach [2] versus incidence approach [2–4], and adjustment for case underreporting; and
- costs subcategories considered, i.e., 1) healthcare components (e.g., medical services, pharmaceuticals, informal caregiving), 2) output/productivity (e.g., decreases in market output or production, home production losses), 3) quality-of-life (e.g., decreases in social role engagement, pain, suffering and loss of enjoyment of life), and 4) program administration (e.g., administration costs of social security payments, accident reporting, compensation payments, and insurance premiums) [13].

Lack of uniformity in national-level data collection across countries is a key reason for less than optimal cross-country comparability of economic burden studies. Inconsistencies can be related to differences in the definition of work-relatedness and/or reporting incentives. Researchers have employed different techniques to address the extent of underreporting in available data. For example, Leigh inflated work injury and disease incidence from the bureau of labour statistics and workers’ compensation programs by 40%, by comparing epidemiological studies with compensation estimates [2]. Safe Work Australia used a work injury survey to adjust work injury underreporting in workers’ compensation data, since it only identifies accepted claims. They also used an attributable fractions approach to adjust for specific types of work disease underreporting [4]. In another study, Kurppa estimated the non-fatal injuries incidence underreporting across European Union (EU) countries by multiplying the registered number of non-fatal injuries of each country by an external coefficient of a benchmark country, i.e., the ratio of fatal to non-fatal injuries [14].

Lack of uniformity across countries can also be associated with differences in record keeping practices. In terms of recorded cases, the EU Labour Force Survey (LFS) [15], records injury cases with at least 1 day lost, while European Statistics on Accidents at Work (ESAW), only records injury cases with more than 3 days lost [16]. Some data sources such as HSE [3] and Safe Work [4] keep records of cases with no days lost. In terms of work diseases, there is much more variation in recorded cases across countries. Some national-level data sources such as Nederlands Centrum Voor Beroepsziekten (NCvB) statistics in The Netherlands [17], records all reported cases of work diseases based on the Dutch occupational health and safety act, while others such as Deutsche Gesetzliche Unfallversicherung (DGUV) in Germany [18] and Työterveyslaitos in Finland only record cases either suspected or recognized [19]. Banca Dati statistica in Italy records work diseases in three categories of reported, definite and indemnified [20].

The aim of our study is to develop a comprehensive framework for estimating the economic burden of work injuries and diseases and implement it for selected EU countries. Five countries were identified as test cases based on data availability and regional representation, namely Finland, Germany, Italy, The Netherlands, and Poland. The estimated economic burdens identified in the study reflect the monetary value of benefits that would be gained by each country if they had no work injuries or diseases. Our study is part of a larger initiative with multiple stages that include plans to estimate the economic burden of work injuries and diseases in 28 EU countries plus Norway and Iceland.

## Methods

### Conceptual framework development

Our framework draws on several bottom-up economic burden studies, specifically Tompa et al. [21], Leigh [2], Safe Work Australia [4], and HSE [3]. We advance previous methodologies on two fronts. Firstly, we include more cost subcategories, such as informal caregiving time, presenteeism (i.e., reduced on-the-job productivity) [11], home production, employer’s friction/adjustment and quality of life costs. Secondly, we address work injury and disease underreporting by considering secondary data drawn from various sources. The methodology is detailed in a funding report by the same author group [22]. We summarize the key components here.

**Fig. 1:**
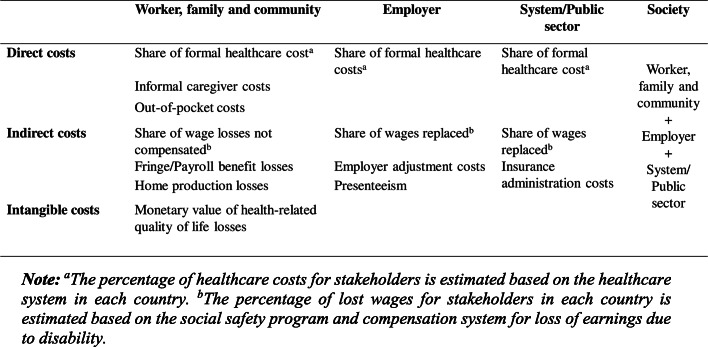
Framework for categorizing the economic burden of work injuries and diseases (adapted from Tompa et al. 2017)

In Fig. 1 we illustrate the cost categories by stakeholders, specifically workers and their families, employers, and system/public sector. We use an estimated approach similar to Safe Work Australia [4], in which costs are based on burdens post-incident. The distribution of these burdens will depend on a country’s social safety net programs, as well as their compensation and healthcare systems. In the Additional File, Table 1, we provide more details on the estimation of burdens by stakeholders. In the estimation of the societal-level burden, we remove transfer payment in the aggregation across stakeholder groups.

### Incidence

#### Injuries

Non-fatal injury incidence for the year 2015 comes from ESAW for injuries with four or more days lost (4–14, 15–90, 90–180, 180–365 days lost and never return) [16] and the ad hoc module of the LFS in 2013 [15] for injuries with three or fewer days. We adjust the injury incidence of Italy and Poland for underreporting using Germany’s fatal to non-fatal injury ratio [7, 13]. We do not adjust the injury incidence for Germany, The Netherlands and Finland, as their fatal to non-fatal ratios are comparable to Kurppa [14] (Additional File Tables 2 and 3).

#### Diseases

We estimate non-fatal diseases, from compensated and non-compensated records (accepted/recognized and suspected) for the reference year of 2015, with some exceptions. Specifically, cancers, musculoskeletal disorders, respiratory and circulatory diseases are based on data from the Institute for Health Metrics and Evaluation (IHME) [23] and adjusted following the under-reporting approach used by Safe Work Australia [4]. For diseases with time loss of two or more days, we estimate severity based on data from the LFS (< 3, 4–30, 30–90, more than 90-days lost, and never return) [24]. For fatal disease incidence we also use IHME [23]. For more details, refer to Tables 4 and 5 in the Additional File.

### Cost categories

Three broad cost categories are considered—direct healthcare, indirect productivity and intangible health-related quality of life costs. In the following sections, we summarize their estimation and our underlying assumptions.

#### Direct costs

We consider four healthcare cost items: 1) formal healthcare; 2) out-of-pocket; 3) informal caregiving time and 4) healthcare administration. We assume a nominal healthcare cost of €100 per case for injuries with three or fewer days lost, similar to HSE [3]. For healthcare costs of injuries with four or more days lost, we use data from the Italian National Ministry of Health, since this was the only country healthcare data available for injuries. We estimate costs for other countries using hospital services adjustment ratio from the International Comparisons of Health Prices and Volumes [25]. We assume the treatment episode for injuries is a maximum of 1 year. For healthcare costs of diseases, we use data from Germany (Destatis), since this was the only healthcare data available for diseases. For other countries, we estimate costs using the same adjustment approach as above. We assume treatment episodes are a maximum of 1 year for hearing disorders, infectious diseases, stomach, liver, kidney or digestive problem, skin problems, headache, and eyestrain. We assume treatment episodes are between 1 to 2 years for cardiovascular disorders, pulmonary disorders, musculoskeletal disorders, stress, depression, anxiety and cancers [3]. We estimate out-of-pocket costs as a percentage of the public sector healthcare costs. For informal caregiving, we assume all cases with less than 6 months of lost time receive one-hour of care per day [21]. For cases with time loss of seven or more months, we do not consider informal caregiving after 6 months. The price weight for caregiver time is based on the wage rate for caregiving services. For healthcare administration costs, we use a percentage of healthcare treatment costs. A summary of the four cost categories are in Table 6 in the Additional File.

#### Indirect costs

We consider six indirect cost items: 1) absenteeism and reduced workability; 2) payroll/fringe benefits; 3) employer adjustment, 4) home production, 5) presenteeism and 6) insurance administration. For cases with a fractional day of time loss, we assume no loss of productivity. We assume that cases with less than 6 months of time loss return to work with no long-run productivity losses. We assume cases with more than 6 months of time loss are permanently impaired, with output losses of 35% continuing till standard retirement age (i.e., age 65) [12]. For fatal cases, we assume losses are from time of death till standard retirement age. We estimate losses based on the difference between the current case experience and the average experience of workers in the population, stratified by country, sex and age bracket, using a human capital approach. For worker earnings, we use data from European Statistical Office (Eurostat) [26], Organisation for Economic Co-operation and Development (OECD) [27, 28], and the European Working Conditions Survey (EWCS) [29]. We add payroll/fringe benefit for Finland of 22%, Germany 23%, The Netherlands 24%, Italy 28%, and Poland 19% based on Eurostat [26]. We include a productivity growth factor of 1% for earnings beyond 2017 and discount all values to calendar year 2015 (our reference year) [21].

We assume that employer adjustment costs are incurred for expenses related to replacing a worker due to injury or disease. For fatal cases, we use 6 months of wages and benefits for adjustment costs [21]. For non-fatal cases with time loss of four or more days, we use the HSE approach [3]. Specifically, we estimate production disturbance costs as 0.5 days times the daily managerial wage and administrative costs as 2.5 h times the wage for clerical staff.

We assume workers off work are not able to fulfil home production tasks. For permanent impairment and fatal cases, we assume home production activities are lost for the remainder of a standard life. For home production losses, we use data on home production time use and the wage rate for domestic services (Table 7 in the Additional File).

We consider presenteeism only for non-fatal cases with time loss of 1 day to 6 months and for cases with time loss of more than 6 months who return to work. For injury cases, we assume presenteeism is relevant only upon return to work, whereas for disease cases, we assume it is relevant before and after work absence. We estimate presenteeism costs based on Schultz et al. [11] (Table 8 in the Additional File).

We estimate insurance administration as a percentage of lost wages. For Finland, Germany and Poland we use 10.1% [30], The Netherlands 11.5% [31] and Italy 12.2% [32].

#### Intangible costs

We estimate health-related quality of life costs using Quality-Adjusted Life Years (QALYs). For temporary and permanent disability cases, we use multipliers identified based on severity, similar to HSE [3]. We only consider QALY losses for injuries and diseases for cases with three or more days lost, including fatal cases, based on sex- and age-based population data on conditional life expectancy. We value a QALY at €41,100 (£30,000) based on the National Institute for Health and Care Excellence (NICE) [33, 34] (Table 9 in the Additional File).

We compare the total economic burden of the five countries in terms of percentage GDP, to ground the burdens in terms of loss to economic production potential at the national level. As World Health Organization guidelines suggest, comparison of the impact of illness by GDP has clear economic meaning at the macroeconomic level [35].

### Sensitivity analysis

We undertake several one-way sensitivity analyses for key parameters. For example, as a lower bound scenario, we consider only compensated disease cases. We consider additional scenarios that address non-fatal injury underreporting. For QALYs, we consider different monetary values, specifically €27,400 and €61,600 (£20,000–£45,000) [33, 34]. We consider two healthcare costs scenarios, a higher and lower one (±80% of healthcare costs in the baseline scenario). We also investigate lower and higher values for informal caregiving time for diseases with permanent disability (183–550 days), wage-replacement rates (70–90%), and earnings losses of cases with permanent disability (33–38%).

## Results

In Table 1 we present the incidence of fatal and nonfatal work injuries and diseases for each of the five countries. The highest number of non-fatal work injuries are for Italy (1,257,987), then Germany (1,158,865), Poland (697,337), The Netherlands (99,880), and Finland (63,407). The highest number of non-fatal, work diseases are for Germany (1,088,793), then Italy (638,448), Poland (454,090), The Netherlands (220,36), and Finland (67,795). Note that the number of cases can vary for multiple reasons including the labour force size of each country.

**Table 1:** Estimated incidence of work injuries and diseases^a^

Countries	Work injuries		Work diseases	
	Non-fatal^b^	Fatal	Non-fatal	Fatal
Finland	63,407 (2602)	35 (1.4)	67,795 (2782)	628 (25.8)
Germany	1,158,865 (2882)	450 (1.1)	1,088,793 (2708)	13,924 (34.6)
The Netherlands	99,880 (1201)	35 (0.4)	220,368 (2649)	3262 (39.2)
Italy	1,257,987 (5600)	543 (2.4)	638,448 (2842)	10,524 (46.8)
Poland	697,337 (4336)	301 (1.9)	454,090 (2823)	4663 (29.0)

^a^incidence are for 2015 or closest year available, ^b^non-fatal injury cases with more than one day lost. Number in parenthesis represents incidence per 100,000 employed persons

In Table 2 we present the economic burden of work injuries and diseases for each country, stratified by direct, indirect and intangible costs. To assist with cross-country comparisons, we provide per case costs (i.e., cost per injury or disease case) and costs as a percentage of country GDP. Indirect costs are the largest cost category (with the exception of Poland). They range from 72% (Finland) to 45% (Poland). Intangible costs are the second-largest category, ranging from 51% (Poland) to 20% (Finland and The Netherlands). Direct costs are the smallest category, ranging from 8% (Finland) to 4% (Poland).

**Table 2:** Estimated total economic burden of work injuries and diseases^a^

Costs	Cases	Direct costs	Indirect costs	Intangible costs	Total costs	Percent of GDP	Per case costs	Per employed person costs
Finland	131,867	M€484 (8%)	M€4362 (72%)	M€1196 (20%)	M€6042	2.9%	€45,816	€2479
Germany	2,262,031	M€10,914 (10%)	M€70,658 (66%)	M€25,557 (24%)	M€107,129	3.5%	€47,360	€2664
The Netherlands	323,544	M€2137 (9%)	M€16,468 (69%)	M€5147 (22%)	M€23,751	3.5%	€73,410	€2855
Italy	1,907,504	M€8491 (8%)	M€58,961 (56%)	M€37,392 (36%)	M€104,844	6.3%	€54,964	€4667
Poland	1,156,394	M€1882 (4%)	M€19,588 (45%)	M€22,311 (51%)	M€43,781	10.2%	€37,860	€2722

^a^all monetary values are in 2015 Euros

The per case costs are highest for The Netherlands (€73,410), then Italy (€54,964), Germany (47,360), Finland (€45,816), and Poland (€37,860). For total costs as a percentage of GDP, the highest is for Poland (10.2%), then Italy (6.3%), Germany and The Netherlands (3.5%), and Finland (2.9%). Costs per employed persons (i.e., cost per each employee in the labour force) are highest for Italy (€4667), then The Netherlands (€2855), Poland (€2722), Germany (€2664) and Finland (2479).

In Table 3 we present the costs by country stratified by 1) worker, family and community, 2) employer, and 3) system/public sector. For comparison purposes, we focus on the percentage of total costs by stakeholders. We find that for all five countries, the highest costs are borne by workers. These costs range from 79% (Poland) to 61% (Germany). For all countries, the second-highest costs are borne by employers. These costs range from 22% (Finland) to 11% (Poland). The lowest costs are borne by the system/public sector, with a range of 19% (Germany) to 10% (Poland). Table 10 in the Additional File provides more granular data for each cost category.

**Table 3:** Economic burden of work injuries and diseases by stakeholders^a^

Country	Worker, family and community	Employer	System/Public sector
Finland	M€3800 (63%)	M€1325 (22%)	M€916 (15%)
Germany	M€64,813 (61%)	M€21,534 (20%)	M€20,782 (19%)
The Netherlands	M€17,235 (73%)	M€3484 (15%)	M€3032 (13%)
Italy	M€70,391 (67%)	M€20,632 (20%)	M€13,821 (13%)
Poland	M€34,421 (79%)	M€5007 (11%)	M€4353 (10%)

^a^all monetary values are in 2015 Euros

In Table 4 we present one-way sensitivity analyses by country. The burden ranges from 0.92 to 3.17% in Finland, 1.02 to 3.85% in The Netherlands, 1.18 to 3.94% in Germany, 4.53 to 7.48% in Italy, and 5.78 to 12.77% in Poland. Sensitivity analyses indicate that injuries and diseases incidence, the value used for a QALY, healthcare costs, earnings losses of cases with permanent disability and informal caregiving time are parameters with the biggest impact on the economic burden.

**Table 4:** Lower and upper bounds of inputs for sensitivity analysis of economic burden of work injuries and diseases^a^

Scenarios		Finland	Germany	The Netherlands	Italy	Poland
Work diseases incidence^b^	Fatal^j^	115;629	2343;13,923	525;3261	1255;10,526	135;4663
	Non-fatal^j^	1776;67,797	36,202;1,088,793	8073;220,368	19,314;638,448	2351;454,090
	GDP (%)	0.92;2.88	1.18;3.52	1.02;3.48	4.53;6.34	5.78;10.18
	GDP change (%)	−68.2;0	−66.5;0	−70.7;0	−28.6;0	−43.2;0
Work injuries incidence with workday lost^c^	Non-fatal (adjustment ratio)	69,748;76,088 (1.1;1.2)	1,274,751;1,390,638 (1.1;1.2)	109,867;119,855 (1.1;1.2)	983,714;1,531,192 (2.9;4.5)	545,300;848,783 (6.2;9.6)
	GDP (%)	2.96;3.03	3.62;3.71	3.55;3.63	5.40;7.29	8.95;11.41
	GDP change (%)	+ 2.57;+ 5.14	+ 2.72;+ 5.44	+ 2.27;+ 4.55	−14.95;+ 14.89	−12.13;+ 12.08
Work injuries incidence without workday lost^d^	Non-fatal	0;97,933	0;1,031,806	0;88,928	0;50,538	0;9363
	GDP (%)	2.88;2.89	3.52;3.52	3.48;3.48	6.34;6.35	10.18;10.18
	GDP change (%)	0;+ 0.26	0;+ 0.03	0;+ 0.04	0;+ 0.02	0;+ 0.02
Monetary value of a QALY^e^	Euro	27,397;61,644	27,397;61,644	27,397;61,644	27,397;61,644	27,397;61,644
	GDP (%)	2.69;3.17	3.24;3.94	3.22;3.85	5.59;7.48	8.45;12.77
	GDP change (%)	−6.60;+ 9.90	−7.95;+ 11.93	−7.22;+ 10.84	−11.89;+ 17.83	−16.99;+ 25.48
Healthcare costs^f^	Range	−0.8;0.8	−0.8;0.8	−0.8;0.8	−0.8;0.8	−0.8;0.8
	GDP (%)	2.79;2.98	3.29;3.75	3.29;3.65	6.03;6.65	10.01;10.34
	GDP change (%)	−3.34;+ 2.28	−6.58;+ 6.45	−5.23;+ 5.13	−4.97;+ 4.87	−1.64;+ 1.61
Earnings losses of permanent disability^g^	Percent	33;38	33;38	33;38	33;38	33;38
	GDP (%)	2.82;2.99	3.46;3.63	3.41;3.60	6.22;6.58	10.00;10.52
	GDP change (%)	−2.00;+ 3.72	−1.61;+ 3.01	−1.89;+ 3.53	−1.96;+ 3.65	−1.78;+ 3.31
Informal caregiving time of diseases with permanent disability^h^	Days	183;550	183;550	183;550	183;550	183;550
	GDP (%)	2.88;2.90	3.52;3.55	3.47;3.52	6.34;6.38	10.18;10.25
	GDP change (%)	−0.02;+ 0.67	−0.02;+ 0.85	−0.03;+ 1.29	−0.01;+ 0.52	−0.01;+ 0.72
Informal caregiving time of injuries with permanent disability^i^	Days	183;550	183;550	183;550	183;550	183;550
	GDP (%)	2.88;2.89	3.52;3.53	3.47;3.49	6.34;6.41	10.17;10.23
	GDP change (%)	−0.02;+ 0.16	−0.02;+ 0.19	−0.06;+ 0.46	−0.14;+ 1.07	−0.06;+ 0.45

^a^all monetary values are in 2015 Euros, ^b^lower and higher incidence of fatal and non-fatal work diseases (adjustment for underreporting), ^c^lower and higher incidence of non-fatal work injuries with more than 3 workdays lost (adjustment for underreporting), ^d^add healthcare costs of non-fatal work injuries with no workday lost, ^e^lower and higher monetary values of a quality-adjusted life years, ^f^lower and higher range of healthcare costs for injuries and diseases, ^g^lower and higher range for earnings losses of cases with permanent disability, ^h^lower and higher range for informal caregiving time for work diseases with permanent disability, ^i^lower and higher range for informal caregiving time for work injuries with permanent disability, ^j^lower bound includes compensated case and higher bound includes compensated case plus specific types of diseases that estimated through attributable fractions approach (same as the baseline)

## Discussion

The economic burden of work injuries and diseases ranges from 2.9 to 10.2% of GDP for Finland and Poland. Excluding intangible costs, it ranges from 2.1 to 5.2% of their GDP, respectively. Across the five countries, in Poland and Italy, the burden is relatively higher compared to Germany, Finland, and The Netherlands. Intangible costs make up a substantial proportion of the costs in these countries, varying from 20 to almost 50%. Even if intangible costs are not included, the cost estimates for Poland and Italy are still relatively higher. This might be partially explained by the sectoral structure for Poland. Specifically, Poland has a large proportion of workers in agriculture where injury and disease risks are high.

An important value of estimates such as those in our study is the ability to compare economic burdens across countries at a point in time. Our estimates of burdens, in terms of percentage of GDP, are higher than what has been previously estimated in the United States (1.8%) [2], and in the United Kingdom (1%) [3], but are within the range estimated in Australia (4.8%) [4] and Singapore (3.2%) [5]. However, in our study, the incidence of work injuries per employed person (non-fatal: 1201-5600; fatal: 0.4–2.4) and diseases per employed person (non-fatal: 26492,842; fatal: 25.8–46.8) are lower than estimated previously for EU countries, i.e., injuries (non-fatal: not available; fatal: 7.6) and diseases (non-fatal: 8465; fatal: 92.8) [1]. The methodological variation among studies is likely one of the key reasons for these differences.

Despite the similarity of the modeling approach taken (i.e., bottom-up), there are still some differences in the cost items that are considered in the total economic burden in different studies. For example, Leigh estimates a lower percentage than we do, but his estimates do not include intangible costs [2], which vary from 20 to 50% of the total costs in our study. Without them, our results are similar to those of Leigh. Australian estimates are higher than that of the United States, at 4.1% of GDP [2, 4]. Given that intangible costs are not included in the Australian estimates, they are higher than most countries in our study.

Differences in the sectoral structure are likely an important part of the variation in the estimations across countries. For instance, agriculture and industry have a higher rate of work injuries than the service sector. In The Netherlands, there is a high percentage of workers in the service sector relative to other countries in our study. For Poland it is the reverse—there are fewer workers in the service sector and more in industry and agriculture. Consequently, we would expect to see a higher work injury and disease burden in Poland and a lower burden in The Netherlands, all else being equal.

Despite the fact that studies frequently use the percentage of GDP to facilitate comparability across countries, there are questions regarding the suitability of this comparison, as some factors can impact the relative magnitudes that are not associated with cross country variations. For example, we used a fixed monetary value for a QALY across the five countries in our study, which makes the percentage GDP of the economic burden relatively higher in countries with a lower GDP. An alternative would be to use distinct values for each country, based on country-specific willingness to pay for a QALY.

Results of our sensitivity analysis provide insight into the extent to which uncertainty of input parameter values plays a role in the variability of the economic burden estimates. The incidence of work injuries and diseases are the most influential parameters for the economic burden magnitudes. In terms of work disease incidence, although national survey data are available, they suffer from underreporting. For example, cases with longer latencies are often not reported and fatal cases are usually excluded. Some insurance systems may influence reporting behaviour, particularly if there are financial incentives to reduced costs. In some systems, there may be legal obligations to report injuries, but obligations may not always be followed in non-insurance-based systems. The sensitivity analysis results provide insights into the possible range of true values of the burdens and identify input data that warrant refinement in data collection systems.

We advance the economic burden methodology through our study on several fronts. In particular, we have included more cost components such as informal caregiving, home production and presenteeism-related costs in our estimates. Other positive features that our study offers are substantial insights into the different costs that drive the total economic burden and estimation of the economic burden borne by different stakeholders. Furthermore, stratification of the results by age-bracket and sex provides valuable information for policymaking to assist with priority setting. Additionally, using a bottom-up approach makes our results more readily comparable with key studies, such as Leigh [2], HSE [3], and Safe Work [4], since this is the approach more commonly used in the occupational health and safety literature. Finally, given the granularity of country-specific data that we use (e.g., working-age structure, labour-market earnings, employment rates, fringe benefits, survival probabilities and healthcare costs), stratified, cross-country comparisons are possible, allowing for a deeper understanding of factors driving the total burden in different countries.

The framework developed in our study can be used to evaluate the economic burdens at a point in time in different countries in the EU and beyond, and over time within countries, conditional on appropriate data being available. This provides an ideal avenue for evaluating progress in reducing burdens within countries and a better understanding of the cross-country differences in the total burdens and the cost components contributing to the burdens.

Despite the many positive features of our study, there are some limitations. We experienced some challenges in securing data for some components of our framework. Firstly, we do not include some fatal work diseases in our estimates (e.g., bladder cancer, digestive diseases, genitourinary diseases, mental disorders, musculoskeletal disorders, and neurological diseases), because IHME 2016 does not have information on their attributable fraction. Secondly, to estimate the productivity losses of permanent disability, we could not identify a source for data on the number of the cases, or proportion of cases that return to work, and the magnitude or proportion of earnings losses associated with permanent impairment. We simply assume cases with lost time of more than 6 months are cases of permanent disability. We also assume that permanently disabled men and women lose a fraction of their earnings, based on a similar study by Tompa et al. [12]

For future studies, better data will need to be gathered for each country for various components of our conceptual framework in order to refine estimates. This is a task for statistical agencies and researchers to focus on going forward. In some cases, the data may exist but are just not readily available for research purposes. For example, for some countries, we did not have access to injury and disease-specific healthcare and social safety net data, though such data likely exist in some form within the administration of healthcare and social safety net programs. Going forward, there needs to be more collaboration to allow for administrative data liberation for research purposes. In general, statistical agencies, policy decisionmakers, program administrators and researchers might work together to build up and harmonize data collection systems across the EU to facilitate future research on economic burdens and economic impacts of prevention efforts in the area of occupational health and safety.

Development of a standardized economic burden methodology is another important target. Some efforts have been made on this front in Australia and elsewhere, and our study contributes to these efforts. Specifically, our study provides an example of how the conceptual underpinnings of economic burden measurement can be applied in relatively unchartered terrain with data from multiple sources. Undoubtedly, more work is needed to develop a standard for use in countries across the EU and elsewhere.

## Conclusion

There is an increasing interest in better understanding the extent of work injuries and diseases and their economic burden to society. Our study focuses on both the conceptual and applied aspects of estimating the economic burden of work injuries and diseases in five EU countries. We advance the methodologies of previous economic burden studies on several fronts. Our study serves as a template for evaluating the societal economic burden of work injuries and diseases in the EU and beyond. The estimated economic burden of work injuries and diseases in the five countries we considered are substantial, despite efforts over the years to reduce adverse workplace exposures. By stratifying the economic burden within each country across key parameters, we identified the most substantial components of the burden. Our work provides important insights for statistical agencies, policy decisionmakers, program administrators and researchers on areas warranting priority attention for policy and research.

## Supplementary Information

**Additional file 1: Table 1.** Percentage of wages lost by stakeholders. **Table 2.** Estimation of fatal and non-fatal work-related injuries. **Table 3.** Adjusted estimation of fatal and non-fatal work-related injuries incidence. **Table 4.** Estimation of work-related non-fatal disease incidence based on different data sources; reference year is 2015 unless sources were unavailable. **Table 5.** Estimation of fatal work-related diseases. **Table 6.** Healthcare costs of work-related injuries (more than 3 days lost) and diseases. **Table 7.** Home production activities time and hourly wage. **Table 8.** Ratio of presenteeism costs to total costs, by type of injuries/diseases. **Table 9.** Health-related quality of life losses due to work-related injuries or diseases. **Table 10.** Costs subsections of work-related injuries and diseases.

## Data Availability

The datasets generated and/or analysed during the current study are available at the following links: European Statistical Office. Persons reporting an accident at work resulting in sick leave by period off work [hsw_ac3]: http://appsso.eurostat.ec.europa.eu/nui/show.do?dataset=hsw_ac3&lang=en. European Statistical Office. Fatal and non-fatal accidents at work, by sex, age groups, injury groups and NACE Rev. 2 economic sectors [hsw_mi07]: http://appsso.eurostat.ec.europa.eu/nui/show.do?dataset=hsw_mi07&lang=en. NCvB statistiek. Nationale Registratie Beroepsziekten: https://beroepsziekten.nl/statistiek-introductie/ncvb-statistiek-nationale-registratie-beroepsziekten. Deutsche Gesetzliche Unfallversicherung. Current figures and long-term trends relating to the industrial and the public sector accident insurers: http://publikationen.dguv.de/dguv/pdf/10002/dguv-statistiken-englisch-web-final.pdf. Finnish Institute of Occupational Health. Occupational diseases in Finland: http://www.julkari.fi/bitstream/handle/10024/131563/Occupational_diseases_2012.pdf?sequence=1. Banca Dati Statistica. Occupational injury and disease statistical database: http://bancadaticsa.inail.it/bancadaticsa/bancastatistica.asp?cod=2. Institute for Health Metrics and Evaluation. Global Burden of Disease Study: http://ghdx.healthdata.org/gbd-results-tool?params=gbd-api-2016-permalink/7193a516026f9a7df17cf73ea9ce3a5d. European Statistical Office. Persons reporting a work-related health problem resulting in sick leave by period off work [hsw_pb3]: http://appsso.eurostat.ec.europa.eu/nui/show.do?dataset=hsw_ac3&lang=en. European Statistical Office. Structure of earnings survey: annual earnings [NACE_R2]: http://ec.europa.eu/eurostat/web/labour-market/earnings/database. Deutsche Gesetzliche Unfallversicherung. Geschäfts- und Rechnungsergebnisse der gewerblichen Berufsgenossenschaften und Unfallversicherungsträger der öffentlichen Hand: https://publikationen.dguv.de/dguv/pdf/10002/gur-2015_web.pdf. Achmea. Annual Report: https://www.achmea.nl/-/media/achmea/documenten/investors/publicaties/2015/achmea-jaarverslag-2015-eng.pdf?la=nl-nl&hash=B1E1FA45230436ACC79E94D369A29BE697025910. Instituto nazionale per l’assicurazione contra gli infortuni sul lavoro. Instituto nazionale Assicurazione Infortuni sul Lavoro workers compensation scheme-Annual financial accounts: https://www.inail.it/cs/internet/home.html.

## Abbreviations

DGUV: Deutsche Gesetzliche Unfallversicherung
ESAW: European Statistics on Accidents at Work
EU OSHA: European Agency for Safety and Health at Work
EU: European Union
Eurostat: European Statistical Office
EWCS: European Working Conditions Survey
GDP: Gross Domestic Product
HSE: Health and Safety Executive
IHME: Institute for Health Metrics and Evaluation
IWH: Institute for Work & Health
LFS: Labour Force Survey
NCvB: Nederlands Centrum Voor Beroepsziekten
NICE: National Institute for Health and Care Excellence
OECD: Organisation for Economic Co-operation and Development
QALYs: Quality-Adjusted Life Years
TNO: The Netherlands Organisation for Applied Scientific Research
VVA: Valdani Vicari & Associati
