# Supplementary material for: Economic burden of work injuries and diseases: a framework and application in five European Union countries
Source: BMC Public Health. 2021 Jan 6;21:49. doi: 10.1186/s12889-020-10050-7 (PMC7789331; doi:10.1186/s12889-020-10050-7)
Supplement: Supplementary file 1 — Additional file 1: Table 1. Percentage of wages lost by stakeholders. Table 2. Estimation of fatal and non-fatal work-related injuries. Table 3. Adjusted estimation of fatal and non-fatal work-related injuries incidence. Table 4. Estimation of work-related non-fatal disease incidence based on different data sources; reference year is 2015 unless sources were unavailable. Table 5. Estimation of fatal work-related diseases. Table 6. Healthcare costs of work-related injuries (more than 3 days lost) and diseases. Table 7. Home production activities time and hourly wage. Table 8. Ratio of presenteeism costs to total costs, by type of injuries/diseases. Table 9. Health-related quality of life losses due to work-related injuries or diseases. Table 10. Costs subsections of work-related injuries and diseases. [file 12889_2020_10050_MOESM1_ESM.docx]

**ADDITIONAL FILE**

**Table 1. Percentage of wages lost by stakeholders**

| **Country** | **Finland**^[1]^ | | | | **Germany**^[2]^ | | | | **The Netherlands**^[3]^ | | | | **Italy**^[4]^ | | | | **Poland**^[5]^ | | | |
| --- | --- | --- | --- | --- | --- | --- | --- | --- | --- | --- | --- | --- | --- | --- | --- | --- | --- | --- | --- | --- |
| **Days lost** | 30 | 180 | PD | F | <30 | 180 | PD | F | <30 | 180 | PD | F | <30 | 180 | PD | F | <30 | 180 | PD | F |
| **Worker** | 0 | 30 | 30 | 35 | 0 | 20 | 20 | 20 | 30 | 30 | 25 | 40 | 40 | 25 | 25 | 30 | 20 | 20 | 20 | 35 |
| **Employer** | 100 | 0 | 0 | 0 | 100 | 0 | 0 | 0 | 70 | 70 | 0 | 0 | 60 | 75 | 0 | 0 | 80 | 0 | 0 | 0 |
| **System/ Public Sector** | 0 | 70 | 70 | 65 | 0 | 80 | 80 | 80 | 0 | 0 | 75 | 60 | 0 | 0 | 75 | 70 | 0 | 80 | 80 | 65 |

*Days lost: 30: less than 30, 180: more than 30 and less than 180 days lost, PD: permanent disability, F: fatal, [1] Kela (2018), Palkkaus (2018), TVK (2018), [2] TK (2018), BGHW (2018), [3] Rijksoverheid (2018), UWV (2018), Letselschade-kenniscentrum (2018), [4] INAIL (2018), Ponzo et al., (2016), GIESSE (2018), [5] Gratka (2018), INFOR (2018).*

**Table 2. Estimation of fatal and non-fatal work-related injuries (2015)**

|  | **Employed population** | **More than 3 days lost** | | **1-3 days lost** | | **No day lost** | | **Fatal cases^[2]^** |
| --- | --- | --- | --- | --- | --- | --- | --- | --- |
| **Country** |  | **Percent^[1]^** | **Count^[2]^** | **Percent^[1]^** | **Count^[3]^** | **Percent^[1]^** | **Count^[3]^** |  |
| Finland | 2,436,800 | 26 | 42,045 | 13 | 21,362 | 61 | 97,933 | 35 |
| Germany^[4]^ | 40,210,900 | 39 | 845,005 | 14 | 313,859 | 47 | 1,031,806 | 450 |
| The Netherlands^[4]^ | 8,318,700 | 39 | 72,829 | 14 | 27,051 | 47 | 88,928 | 35 |
| Italy | 22,464,800 | 76 | 295,156 | 11 | 42,673 | 13 | 50,538 | 543 |
| Poland | 16,083,900 | 84 | 81,850 | 6 | 6,216 | 10 | 9,363 | 301 |

*[1] Eurostat (2018b),[2] Eurostat (2018a), [3] Calculated number using the percentage based on ^[1],[2],^ [4] To estimate the cases with less than 3 days lost for these countries, we used the distribution from Switzerland, as no data were available in the Labour Force Survey (LFS) ad hoc module 2013 (more than 3, 1-3, and no day lost) (Eurostat 2018a; Eurostat 2018b). We choose Switzerland as conservative assumption, as it has one of the lower levels of reported work-related injuries resulting in sick leave, just slightly higher than Scandinavian countries (Kurppa, 2015) and United Kingdom (HSE, 2011).*

**Table 3. Adjusted estimation of fatal and non-fatal work-related injuries incidence (2015)**

| **Country** | **Employed population** | **Non-fatal cases (>1 day lost)^[1]^** | **Fatal cases^[2]^** | **Fatal to non-fatal ratio ×100,000** | **Adjustment ratio for non-fatal cases underreporting**  **baseline^[3]^** | **Adjusted non-fatal cases^[3]^** |
| --- | --- | --- | --- | --- | --- | --- |
| Finland | 2,436,800 | 63,407 | 35 | 55 | 1 (+1.1, +1.2) | 63,407  (69,748- 76,088) |
| Germany | 40,210,900 | 1,158,865 | 450 | 39 | 1 (+1.1, +1.2) | 1,158,865  (1,274,751-1,390,638) |
| The Netherlands | 8,318,700 | 99,880 | 35 | 35 | 1 (+1.1, +1.2) | 99,880  (109,867- 119,855) |
| Italy | 22,464,800 | 337,829 | 543 | 161 | 3.7 (+2.9, +4.5) | 1,257,987  (983,714-1,531,192) |
| Poland | 16,083,900 | 88,066 | 301 | 342 | 7.9 (+6.2, +9.6) | 697,337  (545,300-848,783) |

*[1] Count from Eurostat (2018a), and distribution from Eurostat (2018b).* *Non-fatal injuries with no workdays lost were excluded in the baseline and only used for sensitivity analysis, [2] Eurostat (2018a),* *The table shows a more or less similar fatal to non-fatal ratio in Finland, Germany and The Netherlands, but a much higher ratio (relatively more fatal cases) in Italy and Poland. The higher ratio is probably an indication of a high rate of underreporting (Kurppa, 2015). Kurppa calculated a coefficient ratio of fatal on non-fatal injuries. In the European union-15, 4,011 fatal and 4,048,491 registered non-fatal work injuries occurred in 2005. Thus, for each fatality there were 1,009 registered non-fatal work injuries. Using this ratio, non-fatal injury of Italy and Poland are not placed in the range, [3] To address underoporting issue, we used the Germany ratio of fatal to non-fatal injuries (39) as a baseline ratio to adjust the non-fatal injury case counts of Poland and Italy. We then ran a sensitivity analysis using the lowest (35) and highest (55) fatal to non-fatal injuries ratios, which were for The Netherland and Finland, respectively. We did not change the baseline injury count for Germany, The Netherlands and Finland. For these three countries our baseline estimated nonfatal injuries (more than 3 days lost) is close to Kurppa (2015). Number in parenthesis represents the lower and higher bound for sensitivity anlaysis.*

**Table 4. Estimation of work-related non-fatal disease incidence based on different data sources; reference year is 2015 unless sources were unavailable**

| **Country** | **Finland** | | **Germany** | | **The Netherlands** | | **Italy** | | **Poland** | |
| --- | --- | --- | --- | --- | --- | --- | --- | --- | --- | --- |
| **Type** | **L**^[1]^ | **B**^[1,6]^ | **L**^[2]^ | **B**^[2,6]^ | **L**^[3]^ | **B**^[3,6]^ | **L**^[4]^ | **B**^[4,6]^ | **L**^[5]^ | **B**^[5,6]^ |
| Cardiovascular disorders |  | 988 | 919 | 17,569 | 63 | 3,959 | 156 | 9,146 |  | 7,793 |
| Hearing disorders | 533 | 1,075 | 534 | 1,075 | 2,489 | 6,223 | 1,699 | 5,181 | 169 | 423 |
| Pulmonary disorders | 651 | 4,847 | 751 | 73,695 | 133 | 13,476 | 1,155 | 31,803 | 1,031 | 39,031 |
| Musculoskeletal disorders | 173 | 58,230 | 23,924 | 949,609 | 2,227 | 181,586 | 12,258 | 558,623 | 98 | 397,551 |
| Infectious diseases |  |  | 356 | 717 | 3 | 8 | 5 | 14 | 660 | 1,650 |
| Stomach, liver, kidney or digestive problem |  |  | 356 | 717 | 34 | 85 | 48 | 145 | 5 | 13 |
| Stress, depression, anxiety |  |  | 3,104 | 6,253 | 2,631 | 6,578 | 199 | 607 | 167 | 418 |
| Skin problems | 336 | 944 | 662 | 1,334 | 229 | 573 | 172 | 525 | 93 | 233 |
| Headache, eyestrain |  |  | 4,231 | 8,523 | 10 | 25 | 33 | 101 | 10 | 25 |
| Other not elsewhere mentioned | 83 | 329 | 1,364 | 2,748 | 254 | 635 | 2,673 | 8,154 | 118 | 295 |
| Nasopharynx cancer |  |  |  | 2 |  |  | 917 | 1 |  |  |
| Larynx cancer |  | 17 |  | 504 |  | 127 |  | 741 |  | 149 |
| Tracheal, bronchus and lung cancer |  | 760 |  | 17,450 |  | 5,062 |  | 14,836 |  | 3,722 |
| Non-melanoma skin cancer |  | 334 |  | 3,577 |  | 617 |  | 3,451 |  | 1,540 |
| Breast cancer |  | 52 |  | 916 |  | 209 |  | 465 |  | 199 |
| Ovarian cancer |  | 32 |  | 515 |  | 112 |  | 504 |  | 100 |
| Bladder cancer |  | 74 |  | 1,628 |  | 424 |  | 2,375 |  | 643 |
| Mesothelioma |  | 108 |  | 1,876 |  | 646 |  | 1,719 |  | 279 |
| Leukemia |  | 5 |  | 80 |  | 25 |  | 54 |  | 27 |
| Other Neoplasms |  |  |  | 4 |  | 1 |  | 2 |  | 1 |
| **Total** | **1,776** | **67,797** | **36,202** | **1,088,793** | **8,073** | **220,368** | **19,314** | **638,448** | **2,351** | **454,090** |

*L: Low scenario; i.e. only includes compensated cases. B: Baseline scenario; i.e. compensated and non-compensated cases with the exceptions for cancers, circulatory diseases, respiratory diseases, and musculoskeletal diseases that were estimated using attributable fractions* *from the Burden of Disease (BoD) study 2016 in the IHME (2016), [1] Työterveyslaitos (2012), [2] DGUV Statistics (2013), [3] NCvB statistiek (2015), [4] Banca Dati Statistica (2015), [5]* *Szeszenia-Dabrowska et al. (2016), [6] IHME (2016).*

**Table 5. Estimation of fatal work-related diseases (2015)**^[1]^

| **Type** | **Finland** | **Germany** | **Netherlands** | **Italy** | **Poland** |
| --- | --- | --- | --- | --- | --- |
| Communicable diseases | 5 | 322 | 66 | 61 | 130 |
| Nasopharynx cancer | 0 | 1 | 0 | 0 | 0 |
| Larynx cancer | 2 | 84 | 15 | 127 | 56 |
| Tracheal, bronchus and lung cancer | 269 | 6,511 | 1,739 | 5,973 | 2,094 |
| Breast cancer | 8 | 167 | 33 | 74 | 50 |
| Ovarian cancer | 29 | 501 | 119 | 483 | 95 |
| Mesothelioma | 99 | 1,660 | 601 | 1,666 | 228 |
| Leukemia | 2 | 39 | 7 | 21 | 12 |
| Other Neoplasms | 1 | 18 | 4 | 8 | 8 |
| Circulatory diseases | 88 | 1,725 | 238 | 551 | 949 |
| Respiratory diseases-COPD | 98 | 2,353 | 411 | 1,012 | 968 |
| Respiratory diseases-Pneumoconiosis | 26 | 512 | 27 | 543 | 61 |
| Respiratory diseases-Asthma | 1 | 31 | 2 | 5 | 12 |
| **Total** | **629** | **13,923** | **3,261** | **10,526** | **4,663** |

*[1] IHME (2016).* *We estimated case counts from IHME 2015 data using attributable fractions for fatal work-related diseases from IHME 2016. However, IHME 2016 does not provide attributable fractions for bladder cancer, digestive diseases, neurological diseases, mental disorders, genitourinary diseases, and musculoskeletal disorders. So, there is no regarding the death count of these diseases.*

**Table 6. Healthcare costs of work-related injuries (more than 3 days lost) and diseases (2015)**

| **Injury** | **Germany**^[2]^ | | **Finland**^[2]^ | **Netherlands**^[2]^ | **Italy**^[1]^ | **Poland**^[2]^ |
| --- | --- | --- | --- | --- | --- | --- |
| Wounds and superficial injuries | €3,849 | | €3,807 | €4,476 | €3,389 | €1,171 |
| Bone fractures | €3,849 | | €3,807 | €4,476 | €3,389 | €1,171 |
| Dislocations, sprains and strains | €912 | | €902 | €1,060 | €803 | €277 |
| Traumatic amputations (loss of body parts) | €22,338 | | €22,095 | €25,980 | €19,667 | €6,799 |
| Concussions and internal injuries | €6,500 | | €6,429 | €7,560 | €5,723 | €1,978 |
| Burns, scalds and frostbites | €7,441 | | €7,360 | €8,654 | €6,551 | €2,265 |
| Poisonings and infections | €1,957 | | €1,935 | €2,276 | €1,723 | €595 |
| Drownings and asphyxiations | €2,174 | | €2,151 | €2,529 | €1,914 | €662 |
| Effects of sound, vibration and pressure | €912 | | €902 | €1,060 | €803 | €277 |
| Effects of temperature extremes, light and radiation | €912 | | €902 | €1,060 | €803 | €277 |
| Shocks | €3,398 | | €3,361 | €3,952 | €2,992 | €1,034 |
| **Disease** | | **Germany**^[3]^ | **Finland**^[2]^ | **Netherlands**^[2]^ | **Italy**^[2]^ | **Poland**^[2]^ |
| Heart disease or attack or other problems in the circulatory system | | €3,336 | €3,300 | €3,880 | €2,937 | €1,015 |
| Hearing problem | | €149 | €147 | €173 | €131 | €45 |
| Breathing or lung problem | | €1,353 | €1,338 | €1,574 | €1,191 | €412 |
| Bone/joint/muscle problem | | €1,206 | €1,193 | €1,403 | €1,062 | €367 |
| Infectious disease (virus/bacteria or other type of infection) | | €171 | €169 | €199 | €151 | €52 |
| Stomach/liver/kidney or digestive problem | | €683 | €676 | €794 | €601 | €208 |
| Stress/depression/anxiety | | €3,953 | €3,910 | €4,598 | €3,480 | €1,203 |
| Skin problem | | €637 | €630 | €741 | €561 | €194 |
| Headache and/or eyestrain | | €1,193 | €1,180 | €1,388 | €1,050 | €363 |
| Other types of health problem | | €1,193 | €1,180 | €1,388 | €1,050 | €363 |
| Nasopharynx cancer | | €11,775 | €11,647 | €13,695 | €10,367 | €3,584 |
| Larynx cancer | | €19,633 | €19,420 | €22,834 | €17,286 | €5,975 |
| Tracheal, bronchus and lung cancer | | €20,403 | €20,181 | €23,730 | €17,963 | €6,210 |
| Non-melanoma skin cancer | | €5,757 | €5,695 | €6,696 | €5,069 | €1,752 |
| Breast cancer | | €6,232 | €6,164 | €7,248 | €5,487 | €1,897 |
| Ovarian cancer | | €8,121 | €8,032 | €9,445 | €7,150 | €2,472 |
| Bladder cancer | | €9,313 | €9,212 | €10,832 | €8,200 | €2,835 |
| Mesothelioma | | €15,704 | €15,533 | €18,264 | €13,826 | €4,779 |
| Leukaemia | | €41,429 | €40,979 | €48,184 | €36,476 | €12,609 |
| Other Neoplasms | | €11,005 | €10,885 | €12,799 | €9,689 | €3,349 |
| **Estimation component of direct costs** | | **Germany** | **Finland** | **Netherlands** | **Italy** | **Poland** |
| Out-of-pocket costs (% healthcare costs) | | 2.9%^[4]^ | 5.4%^[5]^ | 3.9%^[6]^ | 1.9%^[4]^ | 1%^[7]^ |
| Informal caregiving hourly wage | | €12^[8]^ | €19^[9]^ | €25^[10]^ | €19^[9]^ | €5^[11]^ |
| Administration costs (% healthcare costs) | | 2.9%^[12]^ | 5.4%^[13]^ | 3.9%^[14]^ | 1.9%^[15]^ | 1%^[16]^ |
| *[1] Italy National Ministry of Health (2015) (no permanent link available). For estimation per case costs, we divided total healthcare cost of “Italian National Ministry of Health” in each International Classification of Diseases 9/10 by the number of cases served in that ICD9/10 code in a calendar year to estimate per case healthcare costs, [2] Adjustment ratio For Germany 92, Finland 91, The Netherlands 107, Italy 81, and Poland 28 based on International Comparisons of Health Prices and Volumes. Note, the average of all The Organisation for Economic Co-operation and Development (OECD) members is 100 (Lorenzoni et al., 2017), [3] Destatis (2015a), The data is only for hospitalized cases, [4] European Commission (2016), [5] The Commonwealth Fund (2017a), [6] The Commonwealth Fund (2017b), [7] European Commission (2017), [8] Statistics Finland (2018), [9] Destatis (2015b), [10] CBS/StatLine (2017), [11] ZUS (2018), [12] Kela (2015), [13] Techniker Krankenkasse (2016), [14] CZ (2015),[15] OECD (2017), [16] NFZ (2018).* | | | | | | |

**Table 7. Home production activities time and hourly wage**

| **Country** | **Time (hours** **per day)** | | **Hourly wage** | |
| --- | --- | --- | --- | --- |
|  | Men | Women | Men | Women |
| Finland | 2.55^[1]^ | 3.68^[1]^ | €12^[6]^ | €12^[6]^ |
| Germany | 2.15^[2]^ | 3.36^[2]^ | €19^[7]^ | €14^[7]^ |
| The Netherlands | 1.95^[3]^ | 3.45^[3]^ | €25^[8]^ | €25^[8]^ |
| Italy | 2.18^[4]^ | 5.11^[4]^ | €19^[9]^ | €14^[9]^ |
| Poland | 2.65^[5]^ | 4.75^[5]^ | €5^[10]^ | €5^[10]^ |
| *[1] Statistics Finland (2010), [2]Destatis (2015b), [3] SCP (2012), [4] OECD (2016), [5] OECD (2016), [6] Statistics Finland (2018), [7] Destatis (2015b), [8] CBS/StatLine (2017), [9] Same as Germany, as National collective agreements (as of January 2016) for Personal and household services did not report the hour, [10] ZUS (2018).* | | | | |

**Table 8. Ratio of presenteeism costs to total costs, by type of injuries/diseases**

| **Type of disease** | **Presenteeism to total cost ratio**^[1]^ | **Types of injury** | **Presenteeism to total cost ratio**^[1]^ |
| --- | --- | --- | --- |
| Cardiovascular disorders | 0.28 | Bone fractures | 0.17 |
| Hearing disorders | 0.33 | Dislocations, sprains and strains | 0.17 |
| Pulmonary disorders | 0.56 | Traumatic amputations (body parts) | 0.17 |
| Musculoskeletal disorders | 0.60 | Concussions and internal injuries | 0.17 |
| Infectious diseases | 0.14 | Burns, scalds and frostbites | 0.17 |
| Skin problems | 0.33 | Poisonings and infections | 0.17 |
| Stress, depression, anxiety | 0.62 | Drownings and asphyxiations | 0 |
| Stomach, liver, kidney or digestive problem | 0.67 | Effects of temperature extremes, light and radiation | 0 |
| Headache, eyestrain | 0.70 | Effects of sound, vibration and pressure | 0 |
| Other not elsewhere mentioned | 0.33 | Shocks | 0 |
| Wounds and superficial injuries | 0.17 |  |  |

*[1]* *Schultz et al. (2009) estimated presenteeism after returning to work based on total costs (i.e. medical and pharmacy + absenteeism + presenteeism cost) for each injury and disease.*

**Table 9. Health-related quality of life losses due to work-related injuries or diseases**

| Injury severity (days lost) | < 3 | 4- 14 | 15- 90 | 90- 180 | 180- 365 | Never return |
| --- | --- | --- | --- | --- | --- | --- |
| Proxy (lost QALY multiplier)^[1]^ | Minor(1) | F (0.002) | W (0.01) | X (0.03) | S (0.1) | S (0.19) |
| Disease severity (days lost) | < 3 | 4- 30 | 30- 90 | > 90 | | Never return |
| Proxy (lost QALY multiplier)^[1]^ | Minor(1) | F(0.002) | W (0.01) | 2×W (0.02) | | S (0.19) |

*[1] Adopted from HSE (2011), Average health-related quality of life of the general population is estimated using literature, i.e. for Finland (Saarni et al., 2006) , Germany and The Netherlands (Huber et al., 2017), Italy (Scalone et al., 2015), and Poland (Golicki et al., 2017).*

**Table 10. Costs subsections of work-related injuries and diseases (costs are in millions of Euros)**

| **Direct costs** | | | | | | | | | | | | | | | | | | | | |  |
| --- | --- | --- | --- | --- | --- | --- | --- | --- | --- | --- | --- | --- | --- | --- | --- | --- | --- | --- | --- | --- | --- |
| **Country** |  | | | | **Formal healthcare costs** | | | **Healthcare cost Insurance administrative cost** | | | | **Informal caregiver costs** | | | | **Out-of-pocket costs** | | | **Total** | |  |
| Finland | Total | | | | €327 | | | €10 | | | | €87 | | | | €60 | | | €484 | |  |
|  | Percent | | | | 68% | | | 2% | | | | 18% | | | | 13% | | | 100% | |  |
| Germany | Total | | | | €7,230 | | | €388 | | | | €2,343 | | | | €954 | | | €10,914 | |  |
|  | Percent | | | | 66% | | | 4% | | | | 21% | | | | 9% | | | 100% | |  |
| The Netherlands | Total | | | | €1,276 | | | €50 | | | | €623 | | | | €188 | | | €2,137 | |  |
|  | Percent | | | | 60% | | | 2% | | | | 29% | | | | 9% | | | 100% | |  |
| Italy | Total | | | | €5,199 | | | €99 | | | | €2,143 | | | | €1,050 | | | €8,491 | |  |
|  | Percent | | | | 61% | | | 1% | | | | 25% | | | | 12% | | | 100% | |  |
| Poland | Total | | | | €1,113 | | | €11 | | | | €502 | | | | €256 | | | €1,882 | |  |
|  | Percent | | | | 59% | | | 1% | | | | 27% | | | | 14% | | | 100% | |  |
| **Indirect costs** | | | | | | | | | | | | | | | | | | | | |  |
| **Country** |  | | **Market output losses** | | | **Fringe/ Payroll Benefit** | | | **Employer adjust­ment costs** | | **Other Insurance administra­tive cost** | | | **Home produc­tion losses** | | | **Presen­teeism** | | | **Total** |  |
| Finland | Total | | €929 | | | €205 | | | €41 | | €92 | | | €1,213 | | | €351 | | | €4,362 |  |
|  | Percent | | 21% | | | 5% | | | 1% | | 2% | | | 28% | | | 8% | | | 100% |  |
| Germany | Total | | €18,804 | | | €4,417 | | | €773 | | €1,885 | | | €17,519 | | | €7,164 | | | €70,658 |  |
|  | Percent | | 27% | | | 6% | | | 1% | | 3% | | | 25% | | | 10% | | | 100% |  |
| The Netherlands | Total | | €3,818 | | | €921 | | | €159 | | €437 | | | €5,281 | | | €1,987 | | | €16,468 |  |
|  | Percent | | 23% | | | 6% | | | 1% | | 3% | | | 32% | | | 12% | | | 100% |  |
| Italy | Total | | €7,640 | | | €2,121 | | | €483 | | €949 | | | €2,913 | | | €1,841 | | | €58,961 |  |
|  | Percent | | 13% | | | 4% | | | 1% | | 2% | | | 5% | | | 3% | | | 100% |  |
| Poland | Total | | €2,264 | | | €421 | | | €157 | | €218 | | | €2,697 | | | €709 | | | €19,588 |  |
|  | Percent | | 12% | | | 2% | | | 1% | | 1% | | | 14% | | | 4% | | | 100% |  |
| **Intangible costs** | | | | | | | | | | | | | | | | | | | | | |
| **Country** | | **Sex** | | **All cases** | | | **Lost QALYs** | | | **Intangible costs** | | | **Per case cost** | | **Percent of GDP** | | | **Per employed persons** | | | |
| Finland | | Male | | 81,133 | | | 16,315 | | | €670 | | |  | |  | | |  | | | |
|  |  | Female | | 50,734 | | | 12,790 | | | €526 | | |  | |  | | |  | | | |
|  |  | Total | | 131,867 | | | 29,105 | | | €1,196 | | | €9,071 | | 0.57% | | | €491 | | | |
| Germany | | Male | | 1,486,099 | | | 395,630 | | | €16,259 | | |  | |  | | |  | | | |
|  |  | Female | | 775,932 | | | 226,246 | | | €9,298 | | |  | |  | | |  | | | |
|  |  | Total | | 2,262,031 | | | 621,876 | | | €25,557 | | | €11,298 | | 0.84% | | | €636 | | | |
| The Netherlands | | Male | | 194,207 | | | 77,336 | | | €3,178 | | |  | |  | | |  | | | |
|  |  | Female | | 129,337 | | | 47,908 | | | €1,969 | | |  | |  | | |  | | | |
|  |  | Total | | 323,544 | | | 125,244 | | | €5,147 | | | €15,908 | | 0.75% | | | €619 | | | |
| Italy | | Male | | 1,318,116 | | | 661,910 | | | €27,202 | | |  | |  | | |  | | | |
|  |  | Female | | 589,388 | | | 247,971 | | | €10,191 | | |  | |  | | |  | | | |
|  |  | Total | | 1,907,504 | | | 909,881 | | | €37,392 | | | €19,603 | | 2.26% | | | €1,664 | | | |
| Poland | | Male | | 720,740 | | | 348,793 | | | €14,334 | | |  | |  | | |  | | | |
|  |  | Female | | 435,655 | | | 194,115 | | | €7,977 | | |  | |  | | |  | | | |
|  |  | Total | | 1,156,394 | | | 542,908 | | | €22,311 | | | €19,294 | | 5.19% | | | €1,387 | | | |

**ADDITIONAL FILE REFRENCES**

Banca Dati Statistica (2015). Occupational injury and disease statistical database. Available at:http://bancadaticsa.inail.it/bancadaticsa/bancastatistica.asp?cod=2

BGHW (Die Berufsgenossenschaft Handel und Warenlogistik) (2018). Survivors' benefits. Available at:https://www.bghw.de/arbeitnehmer/unsere-leistungen/hinterbliebenenleistungen

CBS/StatLine (2017). Compensation of employees, employment; economic activity NA, 1969-2016. Available at:<https://opendata.cbs.nl/#/CBS/en/dataset/82578ENG/table?ts=1522061414794>

CZ (2016) Financieel jaarverslag 2015. Available at: https://www.cz.nl/~/media/over-cz/financieeljaarverslag-2015-cz-groep.pdf?la=nl-nl&revid=a1645afe-a82f-4fad-9662-d0e48f160122

Destatis (German Federal Statistical Office) (2015a). Krankheitskosten: Deutschland, Jahre, Krankheitsdiagnosen (ICD-10), Geschlecht, Altersgruppen, CSV file of table 23631-0003. Available at: https://www-genesis.destatis.de/genesis/downloads/00/23631-0003_00.csv

Destatis (German Federal Statistical Office) (2015b). Zeitsverwendungserhebung – Aktivitäten in Stunden und Minuten für ausgewählte Personengruppen (2012-2013). Available at:https://www.destatis.de/DE/Publikationen/Thematisch/EinkommenKonsumLebensbedingungen/Zeitbudgeterhebung/Zeitverwendung5639102139005.xlsx

DGUV Statistics (2013). Current figures and long-term trends relating to the industrial and the public sector accident insurers. Available at: http://publikationen.dguv.de/dguv/pdf/10002/dguvstatistiken-englisch-web-final.pdf, pp 56.

European Commission (2016). Finland – Health care & Long-term Care systems. Available at:<https://ec.europa.eu/info/sites/info/files/file_import/joint-report_fi_en_2.pdf>

European Commission (2017). State of Health in the EU Poland. Available at:https://ec.europa.eu/health/sites/health/files/state/docs/chp_poland_english.pdf

Eurostat (2018a). Accidents at work statistics (ESAW). Fatal and non-fatal accidents at work, by sex, age groups, injury groups and NACE Rev. 2 economic sectors [hsw_mi07] (2015), Available at: <http://appsso.eurostat.ec.europa.eu/nui/show.do?dataset=hsw_mi07&lang=en>.

Eurostat (2018b). Persons reporting an accident at work resulting in sick leave by period off work [hsw_ac3] (2013), Accidents at work and other work-related health problems (source LFS). Available at: http://appsso.eurostat.ec.europa.eu/nui/show.do?dataset=hsw_ac3&lang=en.

GIESSE (Risarcimento Danni) (2018). What accidents fall within the workplace accidents? Available at:https://www.giesse.info/it/infortunio-sul-lavoro-chi-paga/

Golicki D, Niewada M. EQ-5D-5L Polish population norms. *AMS* 2017;13:191.

Gratka (2018). When sickness benefit is 100, 80, and when 70 percent salary. Available at: https://gratka.pl/regiopraca/portal/porady/zus/kiedy-zasilek-chorobowy-wynosi-100-80-kiedy-70-proc-pensji

HSE (Health and Safety Executive) (2011). Costs to Britain of workplace fatalities and self-reported injuries and ill health, 2014/2015. 2017. Available at: http://www.hse.gov.uk/research/rrhtm/rr897.htm.

Huber M, Felix J, Vogelmann M, et al. Health-related quality of life of the general German population in 2015: results from the EQ-5D-5L. *Int J Environ Res Public Health* 2017;14:426.

IHME (Institute for Health Metrics and Evaluation) (2016). Rethinking Development and Health: Findings from the Global Burden of Disease Study. Seattle, WA: IHME. IHME Database:(http://ghdx.healthdata.org/gbd-results-tool?params=gbd-api2016permalink/7193a516026f9a7df17cf73ea9ce3a5d).

INAIL (Istituto Nazionale per l'Assicurazione contro gli Infortuni sul Lavoro) (2018). Annuity to survivors. Available at: https://www.inail.it/cs/internet/attivita/prestazioni/prestazioni-economiche/rendita-ai-superstiti.html

INFOR (2018) Jakie świadczenia z tytułu śmiertelnego wypadku przy pracy przysługują członkom rodziny zmarłego?. Available at: https://kadry.infor.pl/kadry/bhp/wypadki_przy_pracy_i_choroby_zawodowe/442130,Jakie-swiadczenia-z-tytulu-smiertelnego-wypadku-przy-pracy-przysluguja-czlonkom-rodziny-zmarlego.html

Kela (Finnish institution in charge of settling benefits) (2018). Sickness benefit. Available at:<https://www.kela.fi/sairauspaivaraha>

Kela T. (2015). verkkopalveluiden suosio jatkaa kasvuaan kohti kevyempää organisaatiota. p 43. Available at:https://www.kela.fi/documents/10180/1169692/Kelan_toimintakertomus_2015.pdf/3b6fd076-a406-48e8-8326-8f00a8384a8a

Kurppa K (2015). Severe under-reporting of work injuries in many countries of the Baltic Sea region: An expolatory semi-quantitative study. Finnish Institute of Occupational Health. Available at: http://www. balticseaosh. net/wp/wp-content/uploads/2015/10/Severe-Under-reporting_ final-report_Kurppa. Pdf

Letselschade-kenniscentrum (2018). Employers liability, compensation for death. Available at:http://www.letselschade-kenniscentrum.nl/schadevergoeding-bij-overlijden.php

Lorenzoni L, Koechlin F. (2017). International comparisons of health prices and volumes: New findings. OECD. Available at: https://www.oecd.org/health/health-systems/International-Comparisons-of-Health-Prices-and-Volumes-New-Findings.pdf

NCvB statistiek (2015). Nationale Registratie Beroepsziekten. Available at:https://beroepsziekten.nl/statistiek-introductie/ncvb-statistiek-nationaleregistratieberoepsziekten).

NFZ (Narodowy Fundusz Zdrowia) (2018). Final financial plan of the National Health Fund for 2015. Available at:http://www.nfz.gov.pl/bip/finanse-nfz/

OECD (The Organisation for Economic Co-operation and Development) (2015). Average annual hours actually worked per worker. OECD Publishing. Available at: [https://stats.oecd.org/index.aspx?DataSetCode=ANHRS#](https://stats.oecd.org/index.aspx?DataSetCode=ANHRS).

OECD (The Organisation for Economic Co-operation and Development) (2016). Time use for home-production related activities per day. Times Use Survey.

OECD (The Organisation for Economic Co-operation and Development) (2017). Tackling Wasteful Spending on Health. Available at: https://www.oecd.org/els/healthsystems/Tackling-Wasteful-Spending-on-Health-Highlights-revised.pdf

Palkkaus (2018). Sick pay. Available at:https://www.palkkaus.fi/cms/article/sairausajan_palkka

Ponzo M, Scoppa V. (2016). Cost-Sharing and Use of Health Services in Italy: Evidence from a Fuzzy Regression Discontinuity Design. Available at:http://ftp.iza.org/dp9772.pdf

Rijksoverheid (2018). When am I entitled to a survivor benefit (Anw benefit)? Available at: https://www.rijksoverheid.nl/onderwerpen/algemene-nabestaandenwet-anw/vraag-en-antwoord/wanneer-heb-ik-recht-op-een-nabestaandenuitkering-anw-uitkering

Saarni SI, Härkänen T, Sintonen H, et al. The impact of 29 chronic conditions on health-related quality of life: a general population survey in Finland using 15D and EQ-5D. *Quality of life Rese* 2006;15:1403-14.

Safe Work Australia (2015). The cost of work-related injury and illness for Australian employers, workers and the community: 2012–13. Safe Work Australia: Canberra, ACT, Australia. Available at: http://www. safeworkaustralia. gov. au/sites/SWA/about/Publications/Documents/940/cost-of-work-related-injury-and-disease-2012-13. docx. pdf. 2015

Scalone L, Cortesi PA, Ciampichini R, et al. Health related quality of life norm data of the Italian general population: results using the EQ-5D-3L and EQ-5D-5L instruments. *Epidemiol Biostat Public Health* 2015;12.

Schultz B, Chen Y, Edington W. The cost and impact of health conditions on presenteeism to employers. *Pharmacoeconomics* 2009;27:365-78.

SCP (The Netherlands Institute for Social Research) (2012). A day with the Dutch – Time use in the Netherlands and fifteen other countries. SCP, The Hague. Available at:https://www.scp.nl/english/Publications/Publications_by_year/Publications_2012/A_day_with_the_Dutch

Statistics Finland (2010). Time Use Survey 2009-2010. Available at:<https://www.stat.fi/tup/tasaarvo/timeuse-and-free-time/index_en.html#domesticwork>

Statistics Finland (2018). The total wage earnings according to the Occupational Classification (AML 2010) per sector and gender.

Szeszenia-Dabrowska N, Wilczynska U. Choroby zawodowe w polsce w 2014 r./occupational diseases in Poland, 2014. Medycyna pracy. 2016 May 1;67(3):327. Available at:http://www.ptmp.ayz.pl/ptmp/wp-content/uploads/2016/11/Choroby-zawodowe-w-Polsce.pdf

Techniker Krankenkasse (2016). Annual financial accounts 2015. Available at: https://www.tk.de/tk/finanzen/einnahmen-ausgaben/945634

The Commonwealth Fund. (2017a). The German Health Care System. Available at:<https://international.commonwealthfund.org/countries/germany/>

The Commonwealth Fund (2017b). The Dutch Health Care System. Available at:https://international.commonwealthfund.org/countries/netherlands/

TK (Die Techniker) (2018). Continued pay insurance. Available at:https://www.tk.de/firmenkunden/service/versicherung/versicherung-faq/entgeltfortzahlungsversicherung-2031358

TVK (Tapaturmavakuutuskeskus) (2018). Compensation for death. Available at:<http://www.tvk.fi/en/workers-compensation-and-insurance/compensation/benefits/compensation-for-death/>

Työterveyslaitos (Finnish Institute of Occupational Health) (2012). Occupational diseases in Finland in 2012. Available at: http://www.julkari.fi/bitstream/handle/10024/131563/Occupational_diseases_2012.pdf?sequence=1

UWV (Werken Aan Perspectief) (2018). I am sick and have an employer. Available at:https://www.uwv.nl/particulieren/ziek/ziek-met-werkgever/inkomen-tijdens-ziekte/detail/loondoorbetaling-tijdens-ziekte

ZUS (Zakład Ubezpieczeń Społecznych) (2018). Minimalne wynagrodzenie za pracę od 2003 r.[Employment, wages and salaries in national economy in first quarter] Available at:http://www.zus.pl/baza-wiedzy/skladki-wskazniki-odsetki/wskazniki/minimalnewynagrodzenie-za-prace-od-2003-r
